# Draft genome sequence of multidrug-resistant *Escherichia coli* MAHK_SCM_BAU_30A strain isolated from a subclinical mastitis cow in Bangladesh

**DOI:** 10.1128/MRA.00713-23

**Published:** 2023-10-27

**Authors:** Tasnia Tabassum Anika, Zakaria Al Noman, Md. Saiful Islam, Nazneen Sultana, Md. Nahid Ashraf, Munmun Pervin, Mohammad Ariful Islam, Mokbul Md. Hossain, Md. Tanvir Rahman, Mohammad Abu Hadi Noor Ali Khan

**Affiliations:** 1 Department of Pathology, Faculty of Veterinary Science, Bangladesh Agricultural University, Mymensingh, Bangladesh; 2 Bangladesh Council of Scientific and Industrial Research, Dhaka, Bangladesh; 3 Department of Microbiology and Hygiene, Faculty of Veterinary Science, Bangladesh Agricultural University, Mymensingh, Bangladesh; 4 Department of Animal Sciences, University of California—Davis, Davis, California, USA; 5 Department of Medicine, Faculty of Veterinary Science, Bangladesh Agricultural University, Mymensingh, Bangladesh; University of Maryland School of Medicine, Baltimore, Maryland, USA

**Keywords:** subclinical bovine mastitis, *E. coli*, whole genome sequencing, MDR, antibiotic resistance genes, virulence factor genes, CRISPR arrays, public health, Bangladesh

## Abstract

This study announces the sequence of a multidrug-resistant *Escherichia coli* MAHK_SCM_BAU_30A strain isolated from bovine subclinical mastitis milk in 2022 in Bangladesh. Our assembled genome had a length of 4,884,948 bp, three plasmids, two CRISPR arrays, five prophages, 51 predicted antibiotic resistance, and 72 predicted virulence factor genes.

## ANNOUNCEMENT

The global public health is at risk due to the widespread application and improper use of antibiotics, resulting in the emergence of antimicrobial resistance in various multidrug-resistant bacterial strains (1). Subclinical mastitis is a prevalent ailment in lactating dairy cows that may lead to diminished production and financial setbacks for farmers. *Escherichia coli* is recognized as the predominant bacterium capable of inducing subclinical mastitis and displaying resistance to antibiotics (2).

Between June and December 2022, milk samples were collected from cattle with subclinical mastitis in Baghabari (24.1369°N, 89.5859°E) within the Sirajganj district of Bangladesh and transported to the laboratory (24.7245°N, 90.4372°E). These samples were placed in nutrient broth (HiMedia, India), incubated at 37°C overnight, and subsequently spread on eosin methylene blue agar (HiMedia, India) media and incubated at 37°C overnight again. The resulting colonies underwent Gram staining and biochemical tests (indole, methyl red, Voges-Proskauer, citrate utilization, and sugar fermentation tests) to isolate *E. coli* (3). *E. coli* identification was accomplished using the matrix-assisted laser desorption ionization time-of-flight mass spectrometry assay (4). Antibiotic resistance was determined through the disk diffusion method (5) and the CLSI guidelines (6). Finally, a multidrug-resistant *E. coli* isolate, showing resistance to at least three antibiotic classes (7), was selected for this study and incubated overnight in nutrient broth (HiMedia, India) at 37°C. DNA was then extracted from the collected broth culture using a DNA mini kit (Qiagen, Hilden, Germany). The DNA concentration and purity were determined using a NanoDrop 2000 UV-Vis Spectrophotometer (Thermo Fisher, Waltham, MA, USA). The Nextera DNA Flex Library Prep Kit (Illumina, San Diego, CA, USA) was used to create the DNA library, and the genome sequencing was conducted on the Illumina NextSeq2000 platform, generating paired-end reads with a length of 2 × 150 bp. The genome assembly was performed using Unicycler v.0.4.9 (8), preceded by trimming the raw paired-end reads (*n* = 2,551,778) using Trimmomatic v.0.39 (9) (with parameters leading: 20, sliding window: 4:20:20, trailing: 20, minlen = 36), with the aim of eliminating Illumina adapters, recognized Illumina irregularities, and phiX reads from the data set, and the quality was assessed using FastQC v.0.11.7 (10). The genome was annotated using PGAP v.3.0 (11). In our assembled genome, plasmids were predicted by PlasmidFinder v.2.1 (12); CRISPR arrays by CRISPRimmunity (13); prophages by PHASTER (14); antibiotic resistance genes (ARGs) by CARD v.3.2.4 (15) and ResFinder v.4.1 (16); virulence factor genes (VFGs) by VFDB (17) and VirulenceFinder v.2.0 (18); pathogenicity index by PathogenFinder v.1.1 (19); sequence type by MLST v.2.0 (20), and metabolic functional features by RAST v.2.0 (21). Default parameters were used for all tools unless specified otherwise.

The genome assembly of *E. coli* MAHK_SCM_BAU_30A strain comprised 128 contigs, featuring a G + C content of 50.69%. It included nine contig L50 with 161,852 bp of *N*_50_ value. The total genome size was 4,884,948 bp with a coverage of 16.35×. Within this genome, a total of 4,898 genes, 4,807 CDS, 78 tRNA genes, three rRNA genes, and 158 pseudogenes were identified. The genome contained two CRISPR arrays (with 10 genes, i.e., *csa3*, *cas2*, *cas1*, *cas6e*, *cas5*, *cas7*, *cse2gr11*, *cas8e*, *cas3*, and *WYL*), five prophages, and three plasmids [IncFIA, IncFIB, and IncFII(pHN7A8)]. Through MLST analysis, our genome was categorized as sequence type ST21, and the PlasmidFinder tool revealed a pathogenicity index of 0.945. Moreover, our genome harbored 51 predicted ARGs and 72 predicted VFGs. In RAST, 384 subsystems with 31% coverage and 2,158 genes were identified in our assembled genome.

## Data Availability

The WGS shotgun analysis of *E. coli* MAHK_SCM_BAU_30A was deposited to GenBank under the accession number JAUBOE000000000. The relevant data, including the raw reads, were also submitted with BioProject accession number PRJNA984821, BioSample accession number SAMN35779352, and SRA accession number SRR24954487. In this paper, the specific version being referred to is identified as JAUBOE000000000.1.
